# Model-based iterative reconstruction with adaptive regularization for artifact reduction in electron tomography

**DOI:** 10.1038/s41598-025-86639-y

**Published:** 2025-02-18

**Authors:** Singanallur Venkatakrishnan, Obaidullah Rahman, Lynda Amichi, Jose D. Arregui-Mena, Haoran Yu, David A. Cullen, Amirkoushyar Ziabari

**Affiliations:** 1 Multi-modal Sensor Analytics Group, Oak Ridge National Laboratory, Oak Ridge, TN 37831 USA; 2 Electron Microscopy and Microanalysis Group, Oak Ridge National Laboratory, Oak Ridge, TN 37831 USA; 3 Nuclear Energy Materials Microanalysis Group, Oak Ridge National Laboratory, Oak Ridge, TN 37831 USA

**Keywords:** Electron tomography, Diffraction, Model-based reconstruction, Artifact reduction, Applied mathematics, Computational science, Materials for energy and catalysis, Techniques and instrumentation

## Abstract

Obtaining high-quality 3D reconstructions from electron tomography of crystalline particles embedded in lighter support elements is crucial for various material systems such as catalysts for fuel cell applications. However, significant challenges arise due to the limited tilt range, sparse and low signal-to-noise ratio of the measurements. In addition, small metal particles can cause strong streaking and shading artifacts in the 3D reconstructions when using conventional reconstruction algorithms due to the presence of Bragg diffraction and the large scattering cross-section difference between the materials of the particles and the background support regions. These artifacts lead to errors in the downstream characterization affecting extraction of critical features such as the size of the metal particles, their distribution and the volume of the lighter support regions. In this paper, we present a two-stage algorithm based on metal artifact reduction, utilizing model-based iterative reconstruction methods with adaptive adjustment of regularization parameters. Our approach yields high-quality 3D reconstructions compared to traditional algorithms, accurately capturing both the metal particles as well as the background support. We demonstrate the effectiveness of our algorithm through simulated and experimental bright-field electron tomography data, showing significant improvements in reconstruction quality compared to traditional methods.

## Introduction

Electron tomography^1–3^ (ET) is an advanced technique used to characterize samples at the nanometer and angstrom scales in 3D. A typical dataset involves tilting a sample around a single axis, making a set of measurements of the scattered electrons using a bright-field (BF) and/or annular dark-field (ADF) detector in a scanning transmission electron microscopy (STEM) mode, pre-processing the measurements, followed by the use of a reconstruction algorithm in order to obtain a 3D volume which is a representation of the scanned sample. Due to mechanical and experimental constraints it is typical to collect only a sparse set of measurements in a limited tilt range (like +/- 70$^{\circ }$) potentially with a low signal-to-noise ratio (SNR), leading to severe artifacts such as streaks, noise and structure elongation (missing wedge artifacts) in the final 3D volume when using baseline reconstruction algorithms.

**Fig. 1:**
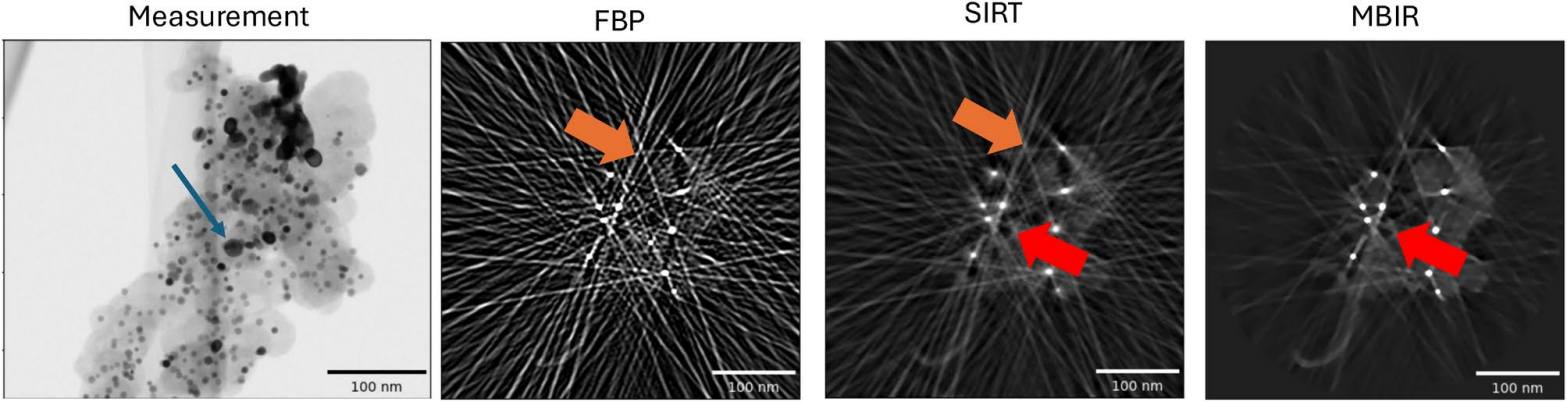
Illustration of the challenges in imaging strongly diffracting metal particles embedded in a lighter support. (**a**) Experimental bright-field STEM data of platinum particles embedded in a carbon support. The image shows a single projection image at a certain tilt angle. The dark regions (indicated with a blue arrow) correspond to particles that strongly diffract the incoming electron beam. The overall data set contains 36 such images in an angular range of $+/- 70^{\circ }$. A single reconstructed cross-section slice from the reconstruction using baseline methods—(**b**) Filtered Back Projection (FBP) (**c**) Simultaneous Iterative Reconstruction Technique (SIRT) and (**d**) Model-based Iterative Reconstruction (MBIR) algorithms. Notice that FBP has the most severe streaking artifacts (shown with orange arrows) due to the low SNR, sparse- and limited-tilt measurement, SIRT suppresses some of these and MBIR has even fewer artifacts. However, even an advanced method like MBIR produces streaks, noise and strong shading (shown with red arrows) which give the appearance of pores around the dense metal particles..

There are special classes of materials in which high-atomic number (Z) crystalline metal particles embedded in a lighter background support material have to be analyzed using ET in order to extract morphological information such as size and distribution of the particles and pores. A typical example is platinum (Pt) fuel cell catalysts where Pt nanoparticles are embedded into a lighter porous carbon (C) support which are used to drive the critical reactions in a hydrogen fuel cell^4,5^. Figure 1 shows a typical measurement from a electron tomographic scan of a Pt-C sample using a BF detector and a cross-section from several typical reconstruction algorithms directly applied to such data. Notice that in addition to the general streaks in several directions that are associated with sparse-tilt data, there is severe streaking in specific directions arising from the Pt particles (orange arrows in Fig. 1) and shading artifacts which make it very challenging to infer important properties such as the size and shape of the particles as well as identification of pores and surface contour of the carbon support. Quantification of these features is necessary to elucidate degradation mechanisms in fuel cells after accelerated stress testing or real-life operation^5,6^ and key to improving the design of durable fuel cells. The artifacts occur because the original measurements are impacted by strong Bragg diffraction^7^ (shown as darker regions in Fig. 1a) from the Pt particles—a non-linear effect which baseline tomographic reconstruction algorithms are not designed to handle—in addition to the typical missing wedge and sparse-tilt related artifacts. While the experiment can be modified to reduce the amount of Bragg diffraction by collecting the incoherent signal using a high-angle annular dark-field (HAADF) detector instead of the BF signal, HAADF is sensitive to highly scattered electrons from heavy atoms (Z-contrast), and may not be suited to resolve all the important features of interest such as the light carbon support regions and identification of the pores. In summary, while ET is a powerful tool for characterization in the material sciences, there are important classes of materials for which the standard reconstruction produces severe artifacts due to limited-angle, sparse-tilt and non-linear measurements.

The choice of reconstruction algorithm plays a critical role in the quality of the final 3D volume and the ability to extract useful information about features of interest using ET. The most popular algorithms used for reconstruction are filtered back projection (FBP)^8^ and the simultaneous iterative reconstruction technique (SIRT). However, it is well known that these methods produce severe artifacts (streaks, particle elongation, noise) for sparse-tilt, limited-angle and low-SNR data sets—which can be typical for ET. The presence of Bragg diffraction from specific material systems such as Pt-C fuel cell catalysts further exacerbates the artifacts (see Fig. 1b,c) and can result in large errors in quantifying parameters such as particle location and size distribution. Specifically, algorithms such as FBP and SIRT implicitly assume that the measurements are related to the unknown 3D object’s attenuation/scatter coefficient via a simple linear projection. However, when imaging special classes of samples these assumptions may not strictly hold, resulting in strong artifacts in the reconstructed volume.

In order to suppress the artifacts from the low-SNR, limited-angle and sparse-tilt data sets, model-based iterative reconstruction (MBIR) algorithms (sometimes referred to as compressed sensing methods)^9–13^ have been developed and shown to produce dramatic improvements in image quality compared to FBP and SIRT methods for ET. MBIR methods obtain tomographic reconstruction by casting the image estimation problem as minimizing a cost function that balances a data-fitting term, that includes the knowledge of the physics of image formation, with a regularization term that encodes prior knowledge of the samples to be imaged. The relative priority for each sets of terms is adjusted via a parameter—often referred to as the regularization parameter—in order to obtain a desirable reconstructed image quality. However, even advanced MBIR algorithms can result in strong artifacts when there are model mismatches/non-linearities^7^ as is the case of strongly diffracting high-Z metal particles embedded in a lighter support material (Fig. 1d). In order to address the challenge of imaging crystalline particles embedded in light support materials, a modified version of the MBIR method was developed^14^ but this method is computationally expensive and requires careful choice of parameters to avoid local minima. Furthermore, the method introduced in^14^ was not evaluated for its abilities to quantify material systems with a high packing density of small particles embedded in lighter support materials.

In this paper we present a new algorithm to obtain high-quality ET reconstruction for samples where there are strongly diffracting particles embedded in lighter support material. Our algorithm takes inspiration from the metal artifact reduction literature^15^, casting the overall reconstruction as a two-stage process. In the first stage, the 3D volume is reconstructed using an MBIR approach with the regularization parameter chosen to easily enable extraction of metal regions using a specifically-designed segmentation algorithm. In the second stage, a modified version of the raw measurements, obtained by interpolating out the projection of the metal mask regions in the original data, is processed by an MBIR method with a different regularization parameter that reconstructs the non-metal regions. The outputs of the two stages are fused to obtain the final reconstruction. We refer to our approach as MBIR-ARAR (adaptive regularization for artifact reduction), a summarized version of which was discussed in a conference abstract^16^. Using simulated and experimental BF-ET data, we demonstrate how we can qualitatively and quantitatively improve reconstructions for ET of crystalline particles embedded in lighter support compared to FBP, SIRT and MBIR methods.

## Overall algorithm

**Fig. 2:**
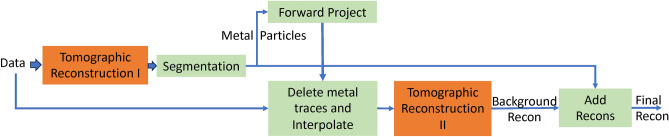
Illustration of outline of metal artifact reduction-based tomographic reconstruction which forms the basis of our approach. The approach involves forming a preliminary reconstruction of the metal/dense components, using it to delete portions of the data impacted by the metal particles, performing a second reconstruction of the non-metal parts and merging the two reconstructions to obtain a final reconstruction. In order to obtain high-quality reconstructions from the sparse-tilt and limited-angle electron tomography data, both the tomographic reconstruction blocks have to produce high quality reconstructions from sparse and low-SNR data. In this work we use model-based iterative reconstruction (MBIR) for each of these blocks with adjustment to the parameters so they are effective in reconstructing the metal particles and the surrounding background in each of the stages respectively.

Our approach for the reconstruction is based on a technique that is particularly popular in the medical computed tomography (CT) literature termed as metal artifact reduction (MAR)^15^. The overall algorithm is summarized in Fig. 2. We first obtain a 3D reconstruction from the measured data. Next, the dense (metal) particles are extracted using an image segmentation routine. Once these are obtained, we forward project the metal particles and determine which locations in the data domain they impact—these regions are then deleted from the original measurements. Since we now have holes in the original data in regions impacted by the dense particles, next we use an interpolation algorithm to fill the gaps in the measured data. Finally, we run a second reconstruction on this metal-subtracted data set to obtain a reconstruction of the non-metal regions (pores, background support etc.). The metal reconstruction from the first stage is then added back to the reconstruction of the non-metal to obtain a final reconstruction.

While this generic pipeline is well-established, adapting it to be able to reconstruct strongly diffracting particles embedded in a light matrix from sparse, limited-angle and potentially low-SNR data poses several challenges such as:

- Baseline algorithms such as FBP, that are the method of choice for the reconstruction due to their low computational complexity, produce very strong artifacts making it challenging to use for our application.
- Fig. 1d shows that even baseline MBIR methods produce streak artifacts for our application. Furthermore, while MBIR techniques can be used to improve over FBP, it is not straightforward to choose parameters to obtain an accurate reconstruction.
- It is challenging to design segmentation algorithms to accurately extract the metal particles from 3D reconstructions with streaking artifacts.
- Finally, each segmentation routine is, in itself, imperfect, therefore the results have to be adjusted to work well with the interpolation algorithms.

Our core contributions are to make use of MBIR algorithms for both reconstruction blocks (orange in Fig. 2) using a software package that makes it intuitive to choose parameters. Furthermore, we designed a segmentation algorithm that can accurately extract the metal particles even from a 3D volume with mild streaking artifacts such as seen in Fig. 1d. We also developed and used an algorithm to accurately delete the regions in the original measurements impacted by metal and fill the gaps using an interpolation algorithm. Next, we present detail of each of these blocks.

### Tomographic reconstruction using MBIR with adaptive regularization

In MBIR, the 3D reconstruction is cast as solving an optimization problem of the form,

1 $$\begin{aligned} \hat{x}(\sigma _y,\sigma _x) \leftarrow \arg \min _{x} \left\{ \frac{1}{2\sigma _{y}^{2}}||y-Ax||_{W}^{2} + \displaystyle \sum _{i,j \in \chi }w_{i,j}\rho \left( \frac{x_i-x_j}{\sigma _x}\right) \right\} \end{aligned}$$

where *A* is the forward projection matrix, *x* is the underlying image reconstruction volume, *y* is the appropriately normalized projection data, *W* is a diagonal inverse noise co-variance matrix, $w_{i,j}$ is a kernel that assigns a lower weight to neighboring voxels that are far apart, $\chi$ is the set of all neighboring voxels in 3D, $\rho$ is a penalty function used to enforce spatial correlations between neighboring voxels, $\sigma _{y}$ is a parameter associated with the noise standard deviation of the measurements, and $\sigma _{x}$ is a regularization parameter used to control the impact of the spatial correlation term relative to the data-fitting term.

In practice, the entries of the noise co-variance matrix are set based on the statistics of the measured data^17,18^. A common choice for transmission tomography (like bright-field electron tomography) is to set the entries of *W* such that $W_{ii}=\exp (-y_i)$ where $y_i$ is the log-normalized measured data. While this has a precise derivation^17,18^ based on the Poisson statistics of the measurements, intuitively such a choice ensures that measurements corresponding to higher counts are weighted higher in the overall cost function optimization. The weighting kernel $w_{i,j}$ is set up so that neighboring voxels that are far apart are weighted less in the computation. In this paper, following previous works, we have set this to be an inverse distance function and assumed a 26 point neighborhood for each voxel in 3D. So $w_{i,j} \propto 1.0/\text {dist}(i,j)$, where $\text {dist}(i,j)$ is the physical distance between neighboring voxels *i* and *j*, with an additional normalization done to scale the entries such that for all the neighbors of voxel *i*, $N_i$, the weights sum to 1, i.e. $\displaystyle \sum _{j \in N_i} w_{ij}=1$.

The role of the penalty function is to induce a certain type of reconstruction. If it is chosen to be a quadratic, $\rho (\Delta )=\Delta ^2$ then the results of running MBIR would be images with smooth edges. If it is set to a $l_{1}$ norm, $\rho (\Delta )=|\Delta |$, then the reconstructions would be flat with sharp edges, giving it a waxy texture. More generally, there is a choice of functions called Generalized Gaussian Markov Random Fields (GGMRF), which has the form $\rho (\Delta )=|\Delta |^p$. The choice of *p* choice depends on what our prior beliefs are about the object itself. In this work, we have chosen the $\rho$ to be the well studied q-Generalized Gaussian Markov Random Field (qGGMRF) model which allows us to set the penalty to $l_2$, $l_1$, and values in between similar to the GGMRF, but is a differentiable function which makes the subsequent optimization problem easier to solve using gradient methods. The values of the q-GGMRF function are chosen so it has a form that the penalty function is approximately $|\Delta |^{1.2}$, a value that has been used in several past works with satisfactory results^10,14,19^. In summary, the specific choice of the $\rho$ function is heuristic, but we have chosen a functional form and parameters that have been demonstrated to produce satisfactory results for a range of tomographic imaging applications.

An often-ignored topic in MBIR, especially in the context of scientific CT^20^, is the selection of the regularization parameters $\sigma _y$ and $\sigma _x$ in order to obtain a satisfactory reconstruction. Despite the existence of methods in the general literature of image reconstruction (see^21^ and references therein), in practice these values are chosen empirically often to obtain a reasonable visual image quality i.e. balancing noise and resolution in the reconstruction. While this approach is common, choosing these parameters is often un-intuitive to the end users of various CT systems. In order to alleviate this issue, we use the svMBIR library^22^. The svMBIR library^22^, one of the first efforts where the package provides automatic reasonable initial estimates for these parameter values using the following relations, is based on two user inputs—the assumed SNR of the raw data ($\sigma _n$) and the desired sharpness in the reconstruction (*S*)

2 $$\begin{aligned} \hat{\sigma }_{y} \leftarrow \sigma _n \sigma _s \end{aligned}$$

where

3 $$\begin{aligned} \sigma _s = \sqrt{\frac{1}{\tilde{M}} \displaystyle \sum _{i=1}^{\tilde{M}}W_{ii}b_{i}y_{i}^{2}} \end{aligned}$$

and $\sigma _x$ is set as

4 $$\begin{aligned} \hat{\sigma }_{x} \leftarrow 0.2*(2^{S}a) \end{aligned}$$

where *a* is a typical value of a voxel in the 3D reconstructed image, estimated as

5 $$\begin{aligned} a = \frac{\displaystyle \sum _{i=1}^{M} b_iW_{ii}y_i}{\tilde{M}N_{c}} \end{aligned}$$

where $N_{c}$ is the number of pixels along the column dimension of the detector and $b_i$ is a binary indicator variable which is one in the non-zero parts of the $y_i$ and $\tilde{M}$ is the number of non-zero entries. The intuition behind setting $\sigma _{x}$ is to reflect the hypothesis that a typical edge in the 3D volume will be about $20 \%$ (0.2) of the average voxel value in the region occupied by the sample. These automated techniques, while empirical, have been observed to provide a desirable reconstruction quality (visually) across a range of conditions (noise levels, number of projections)^23^. Furthermore, svMBIR has a fast implementation that uses multiple CPU cores while also converging fast due to its use of a coordinate descent algorithm^18^ and multi-resolution reconstruction approach.

In our experiments we only adjust the SNR parameter $\sigma _n$ for each reconstruction stage in order to adjust the quality of the reconstruction so that each stage is tuned to a specific task. While this is still done empirically, we have observed that a higher value of SNR for the first stage and a lower value for the second stage yields reasonable overall results for our samples. The higher value of SNR parameter used in the first stage leads to a sharper yet noisier reconstruction but from which we can accurately extract the metal particles using specially designed segmentation algorithms. The lower value of SNR parameter used in the second stage corresponds to a higher regularization and an overall smoother reconstruction of the pores and background support regions. In practice, we determined the specific values of the SNR for the two stages in a subjective manner using values that yielded satisfactory visual quality of the reconstructions. While this seems arbitrary, we emphasize that this is a common practice in electron tomographic reconstructions - from the filter cutoff parameters in FBP to the several parameters associated with newer neural network based algorithms.

### Segmentation of particles from reconstructions with artifacts

**Fig. 3:**
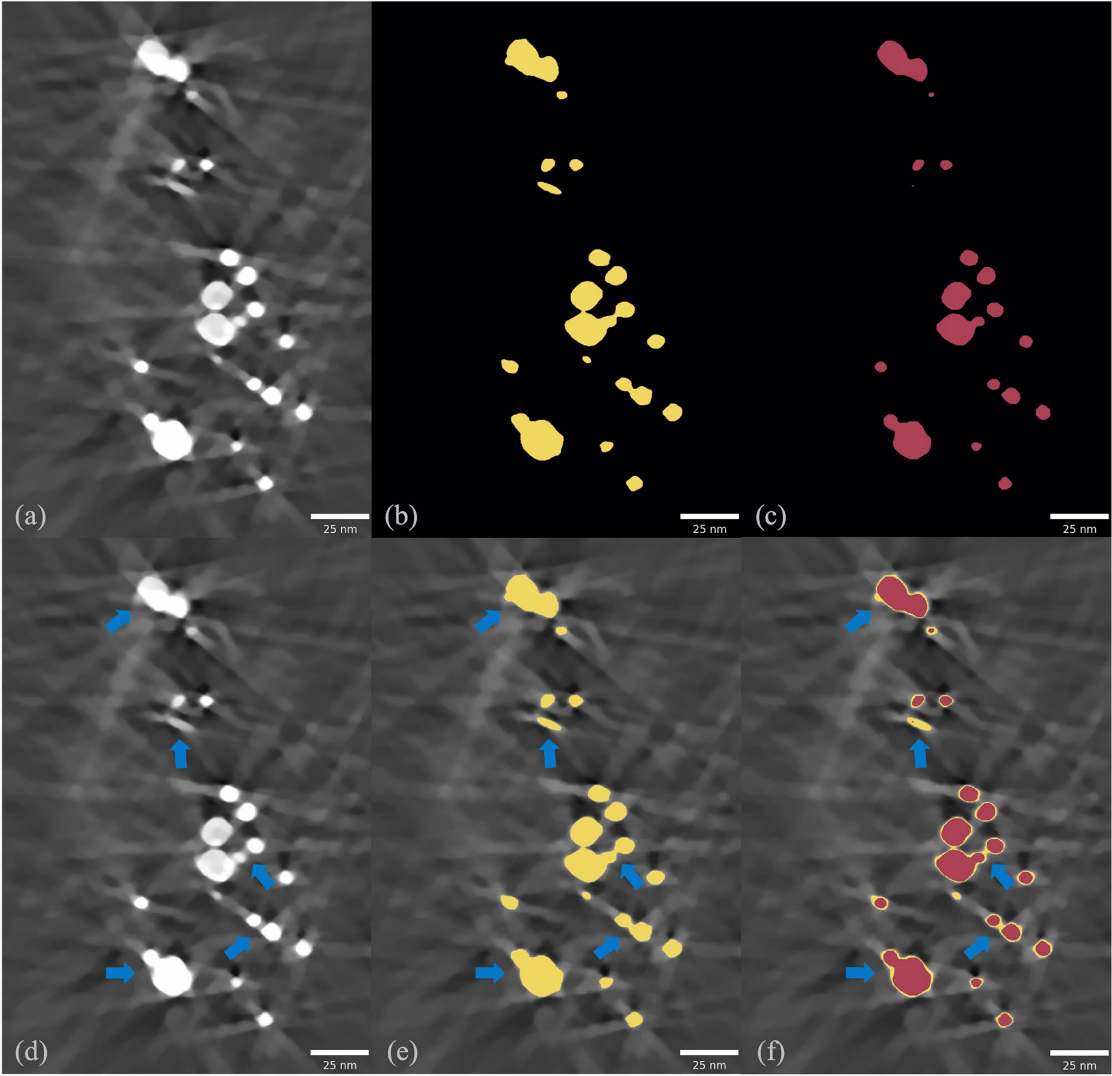
Illustration of the two-stage segmentation to extract the metal particles from an initial reconstruction. The first stage obtains a coarse segmentation while the second stage fine-tunes the output of the first stage to improve the accuracy. (**a**) A cross-section of the 1st stage of reconstruction that shows bright streaks. (**b**) 1st stage of segmentation from multi-Otsu thresholding. (**c**) 2nd stage of segmentation which fine-tunes the segmentation from the 1st stage. (**d**) Same reconstruction cross-section highlighting where streaks may get segmented as metal. (**e**) Layover of 1st stage of segmentation on the reconstruction highlighting that some bright streaks were segmented as metal. (**f**) Layover of 1st and 2nd stage of segmentation on the reconstruction demonstrating the fine-tuning of segmentation from the 1st stage.

One of the strengths of our approach is to be able to segment the metal particles from the background from an initial MBIR despite significant streaking artifacts as shown in Fig. 1d using a two-stage approach. The first stage of the reconstruction can have strong streak artifacts primarily due to Bragg diffraction. Automatic segmentation of the volume into two classes—metal and non-metal—may lead to these bright streaks qualifying as metal thus rendering the segmentation inaccurate. Sometimes the streak from a large particle could be brighter than a small particle, therefore a naive binary threshold may fail (see Fig. 3). In order to address these challenges, we propose a two-stage approach to extract the metal particles. In the first stage, we use a multi-Otsu^24^ algorithm which can be used to separate metal (and some streaks) from the non-metal voxels in the image volume. In the second stage, we label the connected metal regions in the binary volume from the first stage. We then define a rectangular window region around each connected metal region/label and use the Otsu binary thresholding algorithm to separate the metal from the bright streaks in each windowed region. The second stage removes the bright streaks and fine-tunes the metal segmentation because the thresholds are only computed in a small local neighborhood where the streaks have a lower intensity than the actual particle itself. Figure 3 illustrates the working of our segmentation routine by showing a typical reconstructed slice, corresponding initial segmentation and a final refined segmentation.

### Metal deletion and projection interpolation

**Fig. 4:**
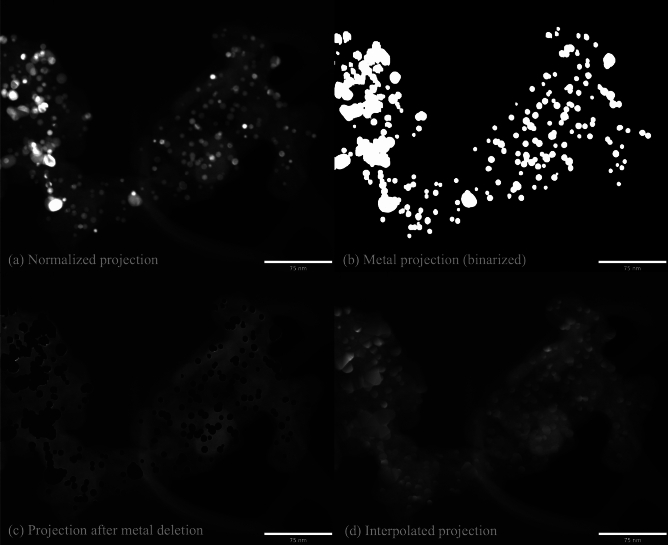
Illustration of the interpolation method used to fill gaps in the projection data resulting from deleting regions impacted by the metal particles. (**a**) A normalized projection image from the tilt series; (**b**) Corresponding metal regions determined from a forward projection of the 3D reconstruction of the metal particles in Fig. 3 in the 1st MBIR-ARAR stage; (**c**) Metal regions from (**b**) subtracted from the original data in (**a**); (**d**) Final interpolated projection image. This process is repeated for all the projections in the tilt series to obtain a new tilt series based on images similar to (**d**). This new tilt series is used to reconstruct the non-metal regions of the sample in the 2nd MBIR-ARAR stage.

The segmented metal particle image from the 2nd stage of segmentation in Fig. 3 is forward projected to get the metal projection as shown in the example in Fig. 4b. Then the pixels where the metal projection is non-zero are deleted in the original normalized projection (Fig. 4a) to obtain images like those in Fig. 4c, and the resulting holes are interpolated from the neighboring pixels that are not affected by metal artifact to obtain the new projection data (Fig. 4d). We used a simple bilinear method (from scipy library^25^) to perform the interpolation. In summary, the metal artifact-affected regions are deleted and contextually replaced with neighboring information. The reconstruction of this interpolated projection in the second stage MBIR provides the 3D volume of regions excluding the metal.

## Results

In this section we demonstrate the strength of the proposed algorithm using simulated and experimental data.

### Simulated data

**Fig. 5:**
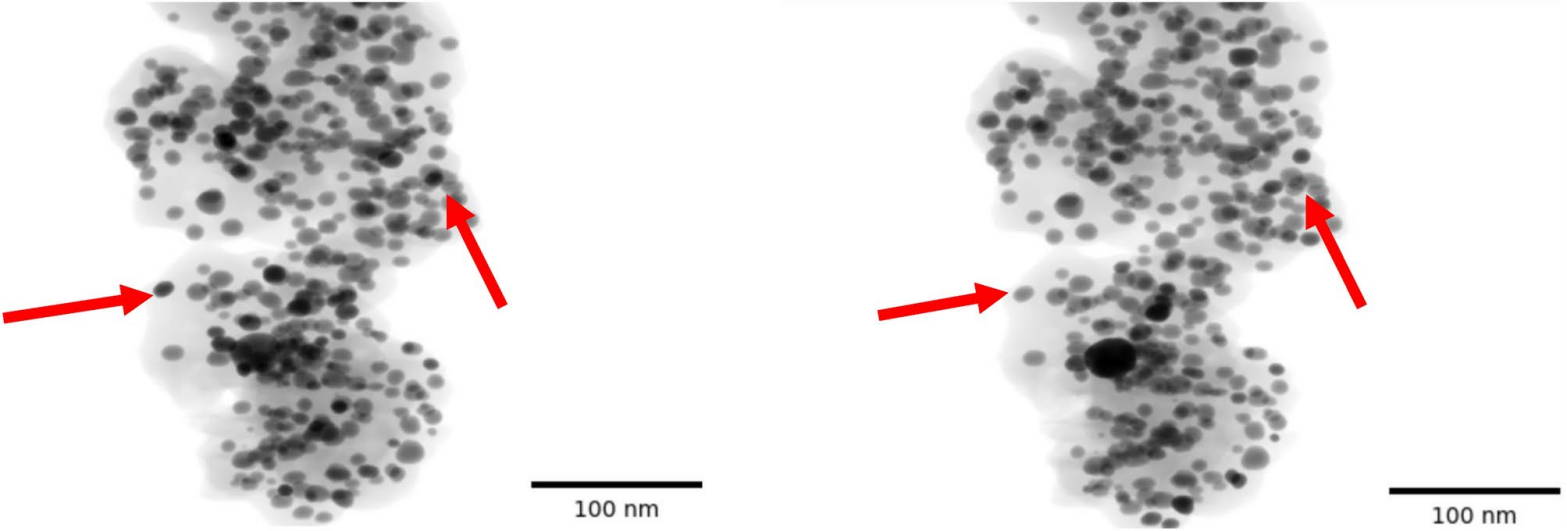
Two subsequent projections from the simulated data of platinum particles embedded in a carbon support. Each image has a size of $800 \times 1200$ pixels. The darker regions (indicated with red arrows) are examples of the regions impacted by particles that strongly diffract the incoming beam leading to a sudden decrease in the transmission signal.

In order to generate simulated BF-STEM data, we use a phantom derived from a previously reconstructed 3D volume (from a HAADF-STEM experiment) containing 3 classes—background support, metal particles and pores. This phantom has 335 metal particles, 198 pores, and has a size of $800 \times 1200 \times 1200$ voxels where 800 is the number of slices perpendicular to the beam direction. The voxel dimension is $0.39 {nm} \times 0.39 {nm} \times 0.39 {nm}$. We assign each of the classes a value for linear attenuation coefficient and project the volume at 36 unique angles in a limited range of +/- $70^\circ$. In order to simulate Bragg diffraction like measurements we use a phenomenological model similar to^14^ that increases the linear attenuation coefficient of the metal particles by a factor of two at a certain pre-determined fraction of all the tilt angles at random. The impact of this sudden increase of attenuation coefficient on the simulated projection data can be seen in Fig. 5 where some regions of the acquired data corresponding to a metal particle are significantly darker in subsequent projections. In our case, the default linear attenuation coefficient values are set to 0 for the pores, $1\times 10^{-3}$ for the carbon support and $2 \times 10^{-2}$ for the metal particles. We emphasize that the model used to simulate Bragg diffraction is heuristic, with the goal being to test the proposed algorithm rather than perfectly match the intensity of the measured data. Furthermore, we simulate noise in each measurement by using a Gaussian approximation to a Poisson random variable as in^14^. The size of the simulated data is 36 projections each of size $800 \times 1200$ pixels.

**Fig. 6:**
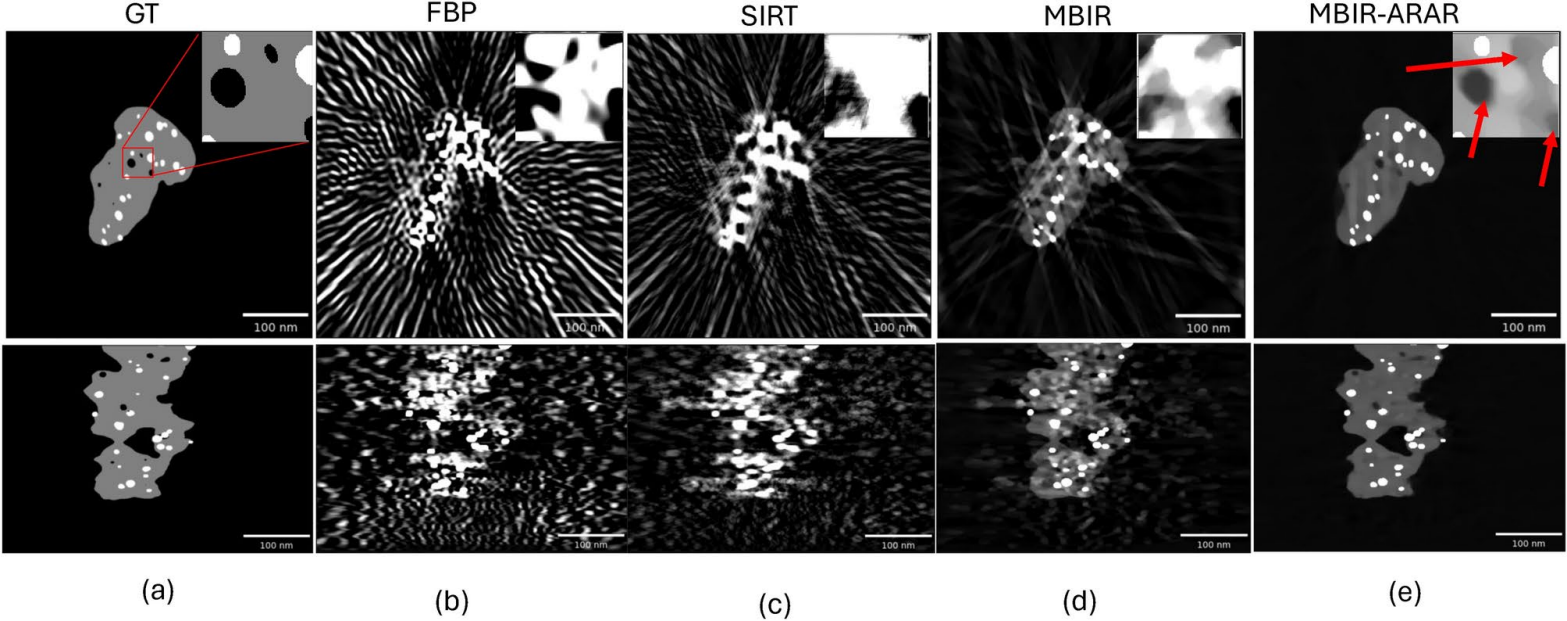
Cross-sections from the 3D reconstructed volume using different algorithms along with an inset of a smaller region of $100 \times 100$ voxels. The top row cross-sections have a size of $1200 \times 1200$ and the bottom row are of size $800 \times 1200$ pixels. The ground truth (GT) images correspond to three intensity levels—0 corresponding to the pores and background (black), 1 corresponding to the carbon support regions (gray), and 3 corresponding to the metal particles (white). All reconstructions are in units of attenuation coefficients with a display window set to $-2\times 10^{-4}$ to $3\times 10^{-3}$. The top row is a slice parallel to the direction of the electron beam and the bottom row shows a slice in one of the perpendicular directions (same as the projections in Fig. 5). The traditional algorithms such as FBP and SIRT produce strong streaking artifacts due to Bragg diffraction in the original data. Even the conventional MBIR contains strong streaking artifacts due to the Bragg diffraction at certain orientations. Furthermore, the conventional MBIR has several false hole-like structures which can lead to an over-estimation of the number of pores and errors in the volume of the carbon support regions. In contrast, the proposed two-stage MBIR-ARAR produces high-quality reconstructions of the support, pores and metal particles compared to all other techniques. The SNR values used to tune the regularizer are 36 dB and 33 dB for the two stages respectively.

Figure 6 shows the reconstructions from the simulated data using different algorithms. The value of the sharpness parameter, *S*, is set to 0 and the SNR parameter values are set to 36 and 33 dB for the two stages of MBIR. The maximum number of iterations for the SIRT and MBIR algorithm is set to 200. For the FBP method we set the filter cut-off parameter to 0.075. The FBP and SIRT algorithms are implemented using the pyMBIR package^26^. Notice that there are strong streaking artifacts in the FBP and SIRT algorithms in addition to the typical limited-angle CT artifacts. The MBIR reconstructions have strong streak artifacts in certain directions that can be attributed to the particles undergoing Bragg diffraction. The proposed MBIR-ARAR method significantly suppresses these artifacts and produces a reconstruction that most closely matches the ground truth. We can visually observe that the regions of the carbon support are accurately reconstructed by the MBIR-ARAR method compared to the other algorithms.

**Table 1:** Analysis of the pores obtained by applying a threshold-based method to the 3D reconstructed volumes from different algorithms. The ground truth contains 198 pores. Notice that obtaining accurate reconstruction of the pores is very challenging using FBP, SIRT and even MBIR as evidenced by the precision, recall and F-score. The MBIR-ARAR reconstruction leads to a significantly better F-score compared to the other methods. However, even the values for the MBIR-ARAR show how challenging it is to detect smaller pores from the sparse-tilt, limited-angle and Bragg diffraction-impacted data.

Metric	FBP	SIRT	MBIR	MBIR-ARAR
# det.	93	11269	126	122
Recall	0.01	0.45	0.13	0.62
Precision	0.02	0.01	0.20	0.99
F-score	0.01	0.02	0.16	0.76

We also quantified the performance of different reconstruction algorithms by their ability to detect pores and parameters associated with the metal particles. Our goal is to evaluate the performance of different algorithms and to determine the overall errors that result in such a challenging setup—limited-angle, sparse-tilt, low-SNR data that is further impacted by Bragg diffraction. First, we quantify the performance of the methods in the reconstruction of the internal pores of the original object. We designed and applied a threshold-based algorithm to obtain a binary segmentation of the 3D volumes from all algorithms followed by a standard connected component analysis in order to identify the pores. Because of the strong artifacts in the standard algorithms, very small (less than 3 voxels) and very large connected components were not included as valid pores. Table. 1 shows an analysis of the pores obtained from the different algorithms along with standard detection metrics—precision, recall, and F-score (harmonic mean of the precision and recall). We identify a pore as being detected if there is significant voxel overlap with the relevant pore in the ground truth. A value of 1 for the precision, recall and F-score identifies a perfect score. The FBP, SIRT and MBIR algorithms perform poorly due to the challenge in obtaining an accurate segmentation of the pores in the ground truth. One indicator is the large number of false positives, which can lead to a large errors in the characterization of porosity if such methods are directly used for 3D reconstruction. The MBIR-ARAR dramatically improves upon the standard algorithms having an F-score of 0.76 compared to values closer to 0 for all other methods. However, we note that the MBIR-ARAR too leads to errors in quantification of the pores in such a challenging set up—mainly due to missed-detections. In particular, we noticed one reason for the higher errors could be missed-detection of almost 70% of smaller pores of size less than 6 voxels. While new segmentation algorithms can be designed to improve the pore detection and therefore the detection metrics, we leave such development to future work.

**Table 2:** Illustration of detection and similarity metrics (rounded to two decimal points) based on reconstructions from different algorithms. There are 335 particles in the ground-truth phantom. The MBIR and MBIR-ARAR methods perform significantly better than FBP and SIRT as can be observed from the F-score. Furthermore, the metal particle size-equivalent diameter histogram from MBIR-ARAR has the lowest EMD suggesting it most closely matches the ground truth.

Particle	FBP	SIRT	MBIR	MBIR-ARAR
#	307	238	334	335
Recall	0.92	0.71	1.00	1.00
Precision	1.00	1.00	1.00	1.00
F-score	0.97	0.83	1.00	1.00
EMD	2434.13	7769.91	166.13	95.30

Next, we quantify the performance of different algorithms on their ability to segment and estimate parameters associated with the metal particles. Table 2 shows the number of detected particles, different detection metrics and the earth movers distance (EMD). The EMD is a measure of the distance between the histogram of size distribution between the ground truth and the reconstructed volumes. In this case, we have used the pyEMD library and binning the data into 50 bins for generating the histogram of the equivalent diameter. We observe that, consistent with the qualitative reconstructions, the values of the EMD is lowest for the proposed MBIR-ARAR method showing strong agreement with the ground truth. Furthermore, the FBP and SIRT approaches under-estimate the number of particles significantly due to the strong streaking artifacts while both the MBIR and MBIR-ARAR result in an accurate computation of the total number of particles—335 in this case. MBIR-ARAR scores a slightly better EMD score than MBIR because of the fine-tuning of the particle segmentation that occurs between stage 1 and stage 2 of the reconstruction.

### Experimental data

The experimental data was acquired manually using a JEM-ARM 200F “NEOARM” (JEOL Inc.) operated at 200 kV. The settings of the microscope were adjusted to obtain measurements of sufficient quality for the Pt-C system being imaged. A 10 $\upmu$m condenser aperture was used to allow a small convergence angle of $\approx 7$ mrad to increase the depth of field. The tilt series were recorded in BF-STEM mode at $\approx 40$ pA electron beam current, 12 cm camera length, with a tilt range from $-75^\circ$ to $75^\circ$ with an increment of $5^\circ$. The pixel resolution is 0.39 nm/pixel. Each projection has a size of $1200 \times 1200$ and 36 projections are used for the reconstruction (leaving out the $+75^{\circ }$ tilt due to certain errors) .

**Fig. 7:**
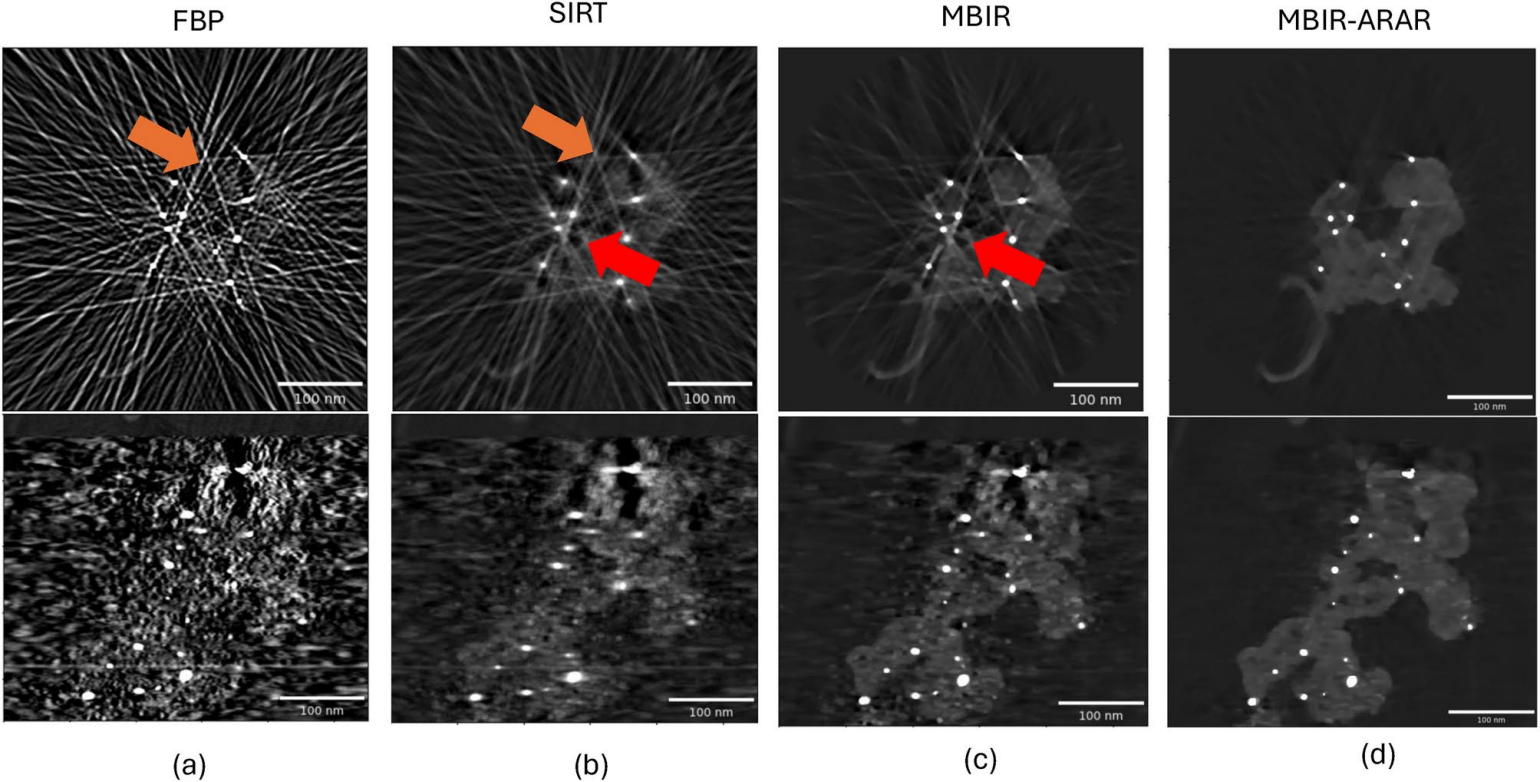
Illustration of various artifacts that occur in electron tomography reconstructions from a limited-angle and sparse-tilt bright-field data set of platinum particles embedded in a carbon support. Two cross-sections from (**a**) FBP (**b**) SIRT-200 iterations (**c**) Conventional MBIR. Notice that the conventional methods like FBP and SIRT produce severe artifacts such as noise, streaks (orange arrows) and halos (red arrows) around the metal regions. The use of MBIR produces sharper reconstructions and helps suppress the artifacts compared to FBP and SIRT for such sparse-tilt data, but we still observe some streaks and dark halos around the dense platinum particles—thereby causing errors in the estimation of various geometric properties such as pore size distribution, characterization of the support etc. Observe that the proposed MBIR-ARAR in (**d**) suppresses streaks and the dark halos around the dense metal regions. The SNR values used to tune the regularizer are 30 and 27 dB for 1st and 2nd stage respectively..

The goal of the study is to characterize the pores, platinum particles and carbon support. The value of the sharpness parameter, *S*, is set to 0 and the SNR parameter values are set to 30 and 27 dB for the two stages of MBIR-ARAR. The maximum number of iterations for the SIRT and MBIR algorithm is set to 200. For the FBP method we set the filter cut-off parameter to 0.15. Figure 7 shows cross-sections from the 3D reconstructions obtained using different algorithms. Similarly to the simulations, the FBP and SIRT algorithms have strong streak artifacts and noise. The MBIR algorithm suppresses these artifacts, but there are still strong streaks due to the Bragg diffraction. However, the proposed MBIR-ARAR method dramatically improves the reconstruction quality and is able to reconstruct the particles, pores as well as the carbon support accurately.

**Fig. 8:**
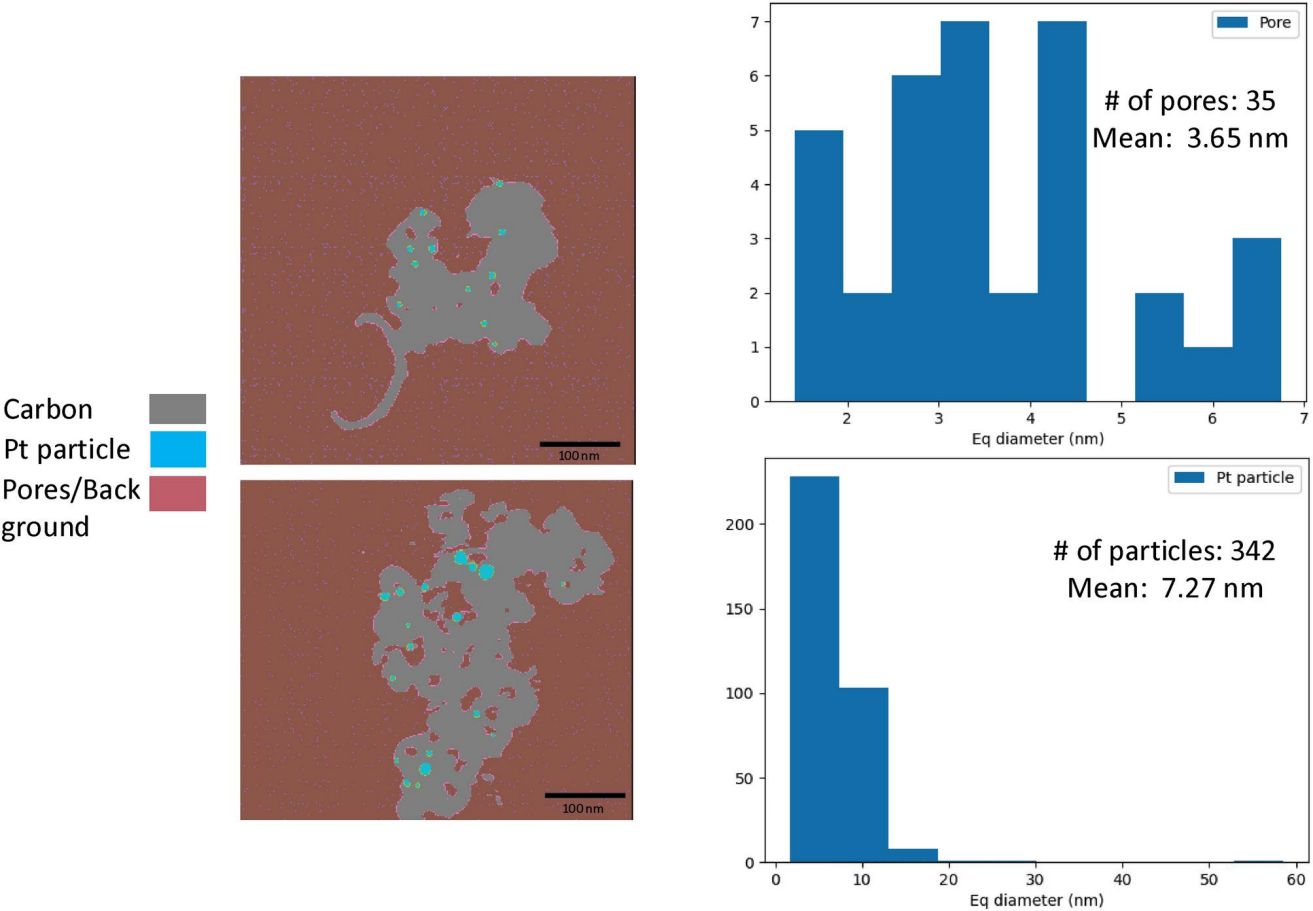
Illustration of the extraction of quantitative information from the MBIR-ARAR reconstruction of the Pt fuel cell catalyst data set. We extract the pores and metal particles using a simple algorithm based on applying a multi-Otsu threshold to the reconstructed intensities. The left panel shows the result of applying the segmentation algorithm to the MBIR-ARAR reconstruction in Fig. 7. The figure also shows the distribution of the equivalent diameter of the pores and metal particles (in nm) along with the number of pores and particles detected by the algorithm. This type of information is critical to further understand the performance of Pt catalysts supported on porous carbon in fuel-cell systems..

Having shown the qualitative improvements of the proposed MBIR-ARAR method, we apply quantitative analysis techniques to the 3D reconstructed volume. Similarly to the simulated data, we first obtain a 3D segmentation of the reconstructed volume. Due to the relative absence of image artifacts, we apply a multi-Otsu threshold algorithm to obtain the location and shapes of pores and metal particles. Figure 8 shows two cross sections of the 3D segmentation along with the corresponding histogram of the pores and platinum particle size distribution. Such information is vital to improve our understanding of the effect of fuel cell operation on Pt nanoparticle growth and structural changes to the carbon support. We emphasize that the use of the MBIR-ARAR provides much more accurate information regarding the pores and particles compared to other baseline algorithms typically used for electron tomography.

## Conclusion

In this paper we presented an algorithm for high-quality electron tomographic reconstruction for material systems comprising dense crystalline material particles embedded in a lighter support. Such reconstructions are very challenging because in addition to the typical limited angle, sparse tilt, and low SNR associated with electron tomographic measurements, they are impacted by the non-linearities due to Bragg diffraction in BF-STEM mode. Our algorithm, MBIR-ARAR, leverages ideas from the metal artifact reduction literature reconstructing the metal and non-metal regions in two stages and fusing them to obtain the final reconstruction. Using simulation data, we demonstrated how our algorithm can dramatically improve the downstream morphological characterization such as the size of particles, the detection of pores and the estimation of the support volume compared to traditional algorithms. However, even when using MBIR-ARAR, the analysis of the reconstruction from simulated data indicates that detecting smaller pores accurately is challenging leading to false positives and missed detections. We apply our method to the Pt fuel cell catalyst system showing its visual superiority over baseline methods and demonstrating how we can easily extract quantitative information about the pore and particles distribution in the sample—which can reveal important information about the behavior of such systems during operation or accelerated stress testing. We note that the validity of MBIR approaches used as a part of our overall algorithm is limited by how far we deviate from the linear projection requirements. Even the first stage of the MBIR produces accurate reconstructions only if the log-normalized measured signal varies almost linearly with the thickness of the sample. This assumption of linearity will not longer be valid for very dense materials at smaller thickness and for lighter materials such as carbon at very large thickness values. Another factor that impacts the performance of the algorithm will be the packing density of the diffracting particles, where a very large value will result in 3D reconstructions with more artifacts. Considering the limitations associated with sample thickness in electron microscopy, including electron scattering, resolution loss, and potential sample damage, careful experimental setup was given allowing for a large depth of field on small slabs< 100 nm at a lower dose to minimizing experimental artifacts and maximizing the resolution of structural features. Finally, we note that designing new reconstruction algorithms that use non-linear physics for modeling Bragg diffraction can further improve the reconstruction quality while also enabling a truly quantitative reconstruction.

## Data Availability

The datasets used in the current study are available from the corresponding author on reasonable request.
